# The Determination of Eight Biogenic Amines Using MSPE-UHPLC-MS/MS and Their Application in Regard to Changes in These Biogenic Amines in Traditional Chinese Dish-Pickled Swimming Crabs

**DOI:** 10.3390/molecules30061353

**Published:** 2025-03-18

**Authors:** Peipei Li, Yu Chen, Junlu Bai, Huicheng Yang, Pengfei He, Junjie Zeng

**Affiliations:** 1 Zhejiang Marine Fisheries Research Institute, Tiyu Road 28, Zhoushan 316021, China; chenxiaoyu2141@163.com (Y.C.); fuzhou.pengfei@163.com (P.H.); junjiezeng123@163.com (J.Z.); 2 College of Food and Pharmacy, Zhejiang Ocean University, 1 South Haida Road, Zhoushan 316000, China; baijunlu@163.com; 3 Zhejiang Marine Development Research Institute, Zhoushan 316021, China

**Keywords:** biogenic amines, pickled swimming crabs, determination, content changes

## Abstract

In this study, a method for the determination of eight biogenic amines (BAs), including tyramine (Tyr), 2-phenylethylamine (2-Phe), histamine (His), tryptamine (Trp), spermidine (Spd), spermine (Spm), cadaverine (Cad), and putrescine (Put), in crab was established using ultra-performance liquid chromatography tandem mass spectrometry (UHPLC-MS/MS), using a magnetic solid-phase extraction (MSPE) pretreatment, without derivatization, and the content changes in regard to these eight biogenic amines in the traditional Chinese dish, pickled swimming crabs, were investigated. The samples were purified via MSPE, using C nanofiber-coated magnetic nanoparticles (Fe_3_O_4_@C-NFs) as sorbents. The experimental variables involved in the MSPE, including the solution pH, adsorption and desorption time, adsorbent usage, and type and volume of the eluent, were investigated and optimized. Method validation indicated that the developed method showed good linearity (R^2^ > 0.995); the average recovery rates were 84.7% to 115%, with the intra-day and inter-day relative standard deviations (RSD, *n* = 6) ranging from 3.7% to 7.5% and 4.2% to 7.7%, respectively. The limit of detection (LOD) and limit of quantification (LOQ) for the eight BAs were 0.1 mg/kg~1.0 mg/kg and 0.3 mg/kg~3.0 mg/kg, respectively. Finally, this method was applied to determine the changes in the eight biogenic amines in pickled swimming crabs (*Portunus trituberculatus*) during storage at 20 °C and 400 BAC. Among the BAs evaluated, Cad, Put, and Tyr were the predominant amines formed during storage. The final content of Cad, Put, and Tyr reached 22.9, 20.1, and 29.0 mg/100 g at 4 °C for 16 d, and 47.1, 52.3, and 72.0 mg/100 g at 20 °C for 96 h, respectively. The results from this study can be used to expand the application range of magnetic materials in biogenic amine pretreatment and to strengthen the quality control of the traditional Chinese dish, pickled swimming crabs.

## 1. Introduction

Biogenic amines (BAs) are basic polar or semi-polar nitrogenous compounds, with a small molecular weight, and they persist in a variety of foods and beverages, such as seafood and its products [1,2], fermented products [3,4,5,6], vegetables [7], and soybean products [8,9,10]. The main BA formation pathway is the decarboxylation of free amino acids by microorganisms, where the α-carboxyl groups are removed from precursor amino acids; the second is the amination or transamination of aldehydes and ketones [11,12,13]. The most commonly generated BAs in food include tyramine (Tyr), 2-phenylethylamine (2-Phe), histamine (His), tryptamine (Trp), spermidine (Spd), spermine (Spm), cadaverine (Cad), and putrescine (Put).

BAs play an essential physiological role in the human body; for instance, they are involved in protein synthesis, intestinal immunity, radical scavenging activity, and brain activity [14,15]. However, high concentrations of BAs in food may cause vascular toxicity and neurotoxicity [16,17]. Among the several types of BAs found in aquatic products, His is the most harmful, followed by Tyr. Both of these substances can result in uncomfortable symptoms, including headaches and irregular blood pressure, and His has the potential to cause neurotoxicity [18]; Cad and Put are produced through the decarboxylation of ornithine and tyrosine, respectively. They contain two amino groups and, while less hazardous, they can, nevertheless, have an impact on health, because they can raise the level of His and Tyr metabolism-related enzymes by blocking their action [18,19]; Trp and 2-Phe are converted from tryptophan and phenylalanine, respectively. Even though they are less poisonous, excessive intake can trigger migraines [20]; Spd and Spm contain multiple amino groups and are catalyzed by various enzymes from putrescine and S-adenosylmethionine. The overaccumulation of Spd and Spm within the body might harm the kidneys and reproductive system [21]. In addition, Spd, Spm, Put, and Cad can form carcinogenic nitrosamines with nitrite [22,23]. Therefore, many countries and organizations have established residue limits for BAs in foods. The European Union (EU) [24] has set an acceptable His level at 100–200 mg/kg in scombroid-like fish; China has set the His limit at 200 mg/kg and 400 mg/kg in fishes with low and high histamine contents, respectively [25]. Establishing a fast and accurate BA detection method is of great significance for ensuring food quality and safety, as well as diagnosing and treating diseases. The predominant amine-forming bacteria in many aquatic products, including fish, crustaceans, and cephalopods, are Gram-negative bacteria. The occurrence of BAs in aquatic products may result either from endogenous amino acid decarboxylases or the growth of microorganisms that possess decarboxylase activity under suitable conditions. Many kinds of BAs are associated with certain types of microbial spoilage in aquatic products, so BAs can be used as important indicators for evaluating the freshness and predicting the shelf life of aquatic products. Hence, research on the change in BA content during the storage of aquatic products is necessary for the supervision of the quality and safety of aquatic products and human health.

Among various detection methods, high-performance liquid chromatography (HPLC) [1,26] and high-performance liquid chromatography–tandem mass spectrometry (HPLC–MS/MS) [27,28] are currently commonly used for their advantages, namely rapid and easy operation, good separation, and high sensitivity and accuracy. Meanwhile, sample pretreatment is also a crucial step for the accurate determination of BAs. Sample pretreatment methods commonly used in this type of study include solid-phase extraction [29,30], matrix solid-phase dispersion extraction [31,32], and ultrasonic-assisted extraction [33,34]. The lack of a conjugated structure in BAs necessitates conventional pretreatment methods to undergo derivatization using reagents, such as o-phthalaldehyde (OPA), dansyl chloride (Dns-Cl), and others, prior to detection. The derivatization reaction often presents challenges, including complex operational procedures, stringent reaction conditions, the instability of derivatized products, and limited method reproducibility. Additionally, in the context of the solid-phase extraction (SPE) methodology, the use of a column necessitates multiple cycles of activation, washing, and elution. Consequently, the pretreatment procedure is time-intensive and hinders the swift analysis and processing of substantial sample quantities.

Magnetic solid-phase extraction (MSPE) has the advantage of achieving rapid phase separation through an external magnetic field, eliminating the step of centrifugal separation, greatly reducing pretreatment time, and simplifying the experimental steps, which has been successfully applied as a new environmentally friendly technique in the extraction and purification of various organic compounds in food [35,36,37,38,39,40], but it has rarely been used for BAs. In this study, MSPE was applied to concentrate trace amounts of BAs in crab samples by using self-made C-nanofiber-coated magnetic nanoparticles (Fe_3_O_4_@C-NFs) as the MSPE adsorbent. The carbon nanofibers consisted of wrapped graphene layers formed into cylinders, which were regarded as quasi-1-D carbon nanomaterials. Due to their structure, comprising six-membered rings and delocalized electrons, carbon nanofibers can adsorb organic molecules, including BAs, through hydrophobic interactions, π-π stacking, hydrogen bonding, and electrostatic and van der Waals forces.

Pickled swimming crab is a special raw food dish, with *Portunus trituberculatus* and rice wine as its main ingredients. This dish is highly popular in coastal regions of China and is considered to be a traditional flavor, due to its traditional salty, tender, and smooth flavor, rich nutritional content, and simple preparation process. The preparation method involves immersing fresh whole crabs in a specific brine solution for a certain period of time. As a minimally processed seafood product, pickled crabs have a longer shelf life compared to fresh crabs and can be easily stored for extended periods. In the Zhejiang coastal region of China, pickled swimming crabs are typically stored at temperatures of 4 to 20 °C. Nevertheless, with the extension of storage time, the accumulation of BAs in pickled crab is inevitable. The contents of biogenic amines in crab with varying storage durations serve as an important indicator of the freshness and edible safety of pickled swimming crabs (*Portunus trituberculatus*). Currently, research on the changes in BAs during the storage of aquatic products primarily focuses on fresh seafood products (Chinese mitten crab, blue swimmer crab, crucifix crab, three spotted crab), with limited studies on pickled swimming crab.

In this study, we developed an efficient and accurate UHPLC-MS/MS method for the analysis of eight BAs in crab samples using Fe_3_O_4_@C-NFs as the MSPE adsorbent. The analytical performance of the method was assessed in terms of linearity, accuracy, precision, the limits of detection and quantification. Subsequently, the established method was applied to investigate the changes in the eight BAs in pickled swimming crabs (*Portunus trituberculatus*) stored at two different temperatures (4 °C and 20 °C). This research demonstrates the feasibility of the non-derivatization pretreatment of BAs using MSPE, streamlining sample preparation and reducing organic solvent use in aquatic products. The findings of this study are valuable for enhancing the quality control of pickled swimming crabs and have practical implications for promoting healthy dietary choices.

## 2. Experimental Section

### 2.1. Materials and Standards

Biogenic amines, including histamine dihydrochloride (His) (C_5_H_9_N_3_⋅2HCl, >99%), 2-phenylethylamine (2-Phe) (C_8_H_11_N, >98%), tryptamine (Trp) (C_10_H_12_N_2_, >98%), spermine trahydrochloride (Spm) (C_10_H_26_N_4_⋅4HCl, >98%), cadaverine (Cad) (C_5_H_14_N_2_, >98%), tyramine hydrochloride (Tyr) (C_8_H_11_NO⋅HCl, >98%), putrescine dihydrochloride (Put) (C_4_H_12_N_2_⋅2HCl, >99%), spermidine trihydrochloride (Spd) (C_7_H_19_N_3_⋅3HCl, >99%) and internal standard (IS) of 1, 7-heptyldiamine, were purchased from Dr. Ehrenstorfer (Augsburg, Germany). Acetonitrile (ACN), methanol (MeOH), formic acid (FA) and n-hexane were HPLC grade and obtained from Merck (Darmstadt, Germany). Sodium hydroxide (NaOH) and trichloroacetic acid (TCA) were obtained from Sinopharm Chemical Reagent Co., Ltd. (Shanghai, China). Ultrapure water for the entire experiment was prepared using a Milli-Q system (Millipore, Bedford, MA, USA). Polytetrafluoroethylene (PTFE) syringe filters (0.22 μm) were supplied by Keyilong (Tianjin, China).

C-nanofiber-coated magnetic nanoparticles (Fe_3_O_4_@C-NFs) were synthesized in the laboratory using a one-pot co-precipitation method, as described in a previous report [41]. In brief, a suitable proportion of FeCl_3_·6H_2_O and CH_3_COONa was dispersed in a solution containing 25 mL deionized water and 50 mL ethylene glycol by stirring. After ultrasonic treatment, an appropriate amount of C-nanofibers was evenly dispersed in the above solution for 1 h. The mixture was then transferred into a stainless steel autoclave and heated at 180 °C for 10 h. Fe_3_O_4_@C-NFs were obtained by washing with deionized water and ethanol several times, followed by drying at 70 °C under vacuum.

Individual stock standard solutions of each BA standard substance and the IS (1, 7-diaminoheptane) were prepared by accurately weighing the respective substances and dissolving them in deionized water. The concentration of each stock solution was about 500 mg/L, calculated according to the respective hydrochloride and purity. The solutions were stored in the dark at 4 °C.

The mixed standard solution was prepared by appropriately diluting aliquots of each standard stock solution with a 75% acetonitrile aqueous solution, resulting in a concentration of 2.0 mg/L. The IS solution was diluted to 10.0 mg/L. A series of mixed standard working solutions were prepared at seven concentrations (5 μg/L, 20 μg/L, 50 μg/L, 200 μg/L, 500 μg/L, 750 μg/L, and 1000 μg/L) with 100 μg/L of IS by further diluting the mixed standard solution with 75% acetonitrile aqueous solution before use. These solutions were also stored in the dark at 4 °C.

### 2.2. UPLC-MS/MS Parameters

Chromatography was performed using Waters Acquity UPLC^TM^ system (Waters, Milford, MA, USA) coupled with Quattro Premier XE Micromass triple-quadrupole mass spectrometer (Waters, Manchester, UK). Chromatographic separation was achieved on a Waters ACQUITY HSS T3 column (2.1 × 100 mm, 1.8 μm particle size) (Waters, Milford, MA, USA) and performed using a binary gradient mobile phase consisting of 5 mmol/L aqueous solution of ammonium acetate (A) and acetonitrile (B) at a flow rate of 0.3 mL min^−1^. The gradient was designed as follows: 0~1.0 min, 100% A; 1.0~5.0 min, 100–50% A; 5.0~5.1 min, 50–5% A; 5.1~6.5 min, 5% A; 6.5~6.6 min, 5–100% A; 6.6~9.0 min, 100% A. The temperature of column and the autosampler was set at 35 °C and 10 °C, respectively. Injection volume was 10 μL, including a needle wash function. MassLynx software 4.1 was used for instrument control and efficient data acquisition.

The mass spectrometer (MS) was operated in positive ion mode with electrospray ionization (ESI). The optimized MS/MS parameters were as follows: cone and desolvation gas: nitrogen (99.9% purity); collision gas: argon (99.9999% purity); source temperature: 150 °C; desolvation temperature: 600 °C; capillary voltage: 3.5 kV; cone gas flow: 50 L·h^−1^; desolvation gas flow: 600 L·h^−1^.

### 2.3. Samples Preparation

*Portunus trituberculatus* was captured from Zhoushan sea area of China. The freshly caught crabs were cleaned, soaked in a 1:30 salt water solution for marination, and then processed into pickled crabs. The samples were divided into two groups and stored at (4 ± 1) °C in a refrigerator and (20 ± 1) °C in a constant temperature incubator, respectively. Under the 4 °C storage conditions, samples were collected at 0, 2, 4, 6, 8, 10, 12, 14 and 16 days after storage. Under the 20 °C conditions, samples were collected at 0, 12, 24, 36, 48, 60, 72, 84 and 96 h. Six parallel samples were collected at each time point, and the edible portions were cut into small pieces no larger than 0.5 cm × 0.5 cm × 0.5 cm. After sampling, the six parallel samples were combined, homogenized, and temporarily frozen, then stored at temperature below −18 °C until the biogenic amine content could be measured within one day. 

### 2.4. Sample Pretreatment

**Figure 1:**
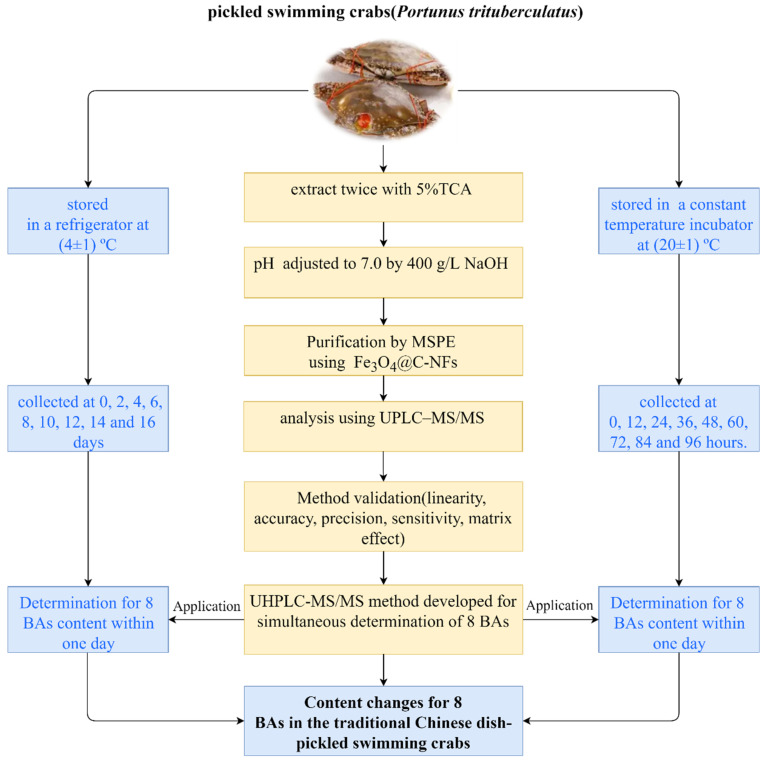
The schematic diagram of the entire procedure in this study.

Further, 2.00 ± 0.01 g homogenized samples were accurately weighed and placed into a 50 mL polypropylene centrifuge tube. Then, 10 mL 5% TCA solution was added and the mixture was homogenized using a vortex mixer for 2 min. Subsequently, the sample was centrifuged at 8000 rpm for 5 min at 4 °C. The supernatant was collected and the residue was re-extracted with an additional 10 mL 5% TCA solution. The two extracts were combined and the pH was adjusted to 7.0 by adding a moderate volume of 400 g/L NaOH solution. The supernatants were subsequently diluted to 50 mL with ultrapure water. Next, 5 mL of the extract was transferred to a glass tube, and 5 mL n-hexane was added to remove remaining fat. Subsequently, 10 mg carbon nanofiber-coated magnetic nanoparticles (Fe_3_O_4_@C-NFs) were added to the extract for the adsorption of BAs under ultrasound for 2 min. The supernatant was discarded with the help of an external magnet. After rinsing with ultrapure water, BAs were eluted from the nanoparticles using 4 mL 5% formic acid-acetonitrile solution via ultrasound for 2 min. The eluent was then dried with nitrogen gas in a 40 °C water bath. The resulting residue was dissolved in 1.0 mL of 10% acetonitrile water solution, filtered through a 0.22 μm filter membrane, and transferred to vials for analysis using UPLC–MS/MS. A schematic flow diagram illustrating the experimental process is depicted in Figure 1.

### 2.5. Statistical Analysis

All stored pickled crab samples were carried out with six replicates and expressed as means ± standard deviations (SDs). Statistical analysis were performed at *p* < 0.05 using SPSS 20.0 software package (SPSS Inc., Chicago, IL, USA). Significant differences between groups at *p* < 0.05 were analyzed using analysis of variance (ANOVA) followed by Duncan’s least significant test.

## 3. Results and Discussion

### 3.1. Optimization of Ultra-Performance Liquid Chromatography–Tandem Mass Spectrometry Conditions

Based on the chemical structure and properties, the BAs and the IS could easily form [M+H]^+^ in positive electrospray scanning ion (ESI^+^) mode. Acquisition parameters were optimized by injecting the individual standard solutions (1.0 μg/mL) of eight BAs into a mass spectrometer with a peristaltic pump at a flow rate of 10 μL/min. The protonated molecular ions [M+H]^+^ were chosen as parent ions; capillary voltage and cone voltage were optimized to achieve maximum intensity. The secondary MS scan was performed using [M+H]^+^ by optimizing the collision gas and collision voltage. Fragmentation patterns were observed, with the loss of one ammonia molecule resulting in the [M+H−NH_3_]^+^ fragment for His, Put, Tyr, 2-Phe and Trp, and the loss of two ammonia molecules producing the [M+H−2NH_3_]^+^ fragment (*m*/*z* 112) for Spd, while the [M+H−(CH_2_)_3_N_2_H_4_−NH_3_]^+^ fragment (*m*/*z* 129.00) for Spm. According to the European Commission Decision 2002/657/EC, two main characteristic fragment ions with strong abundance and the least interference were selected as the qualitative and quantitative ions, respectively. The detailed optimized parameters and MRM transitions are presented in Table 1.

**Figure 2:**
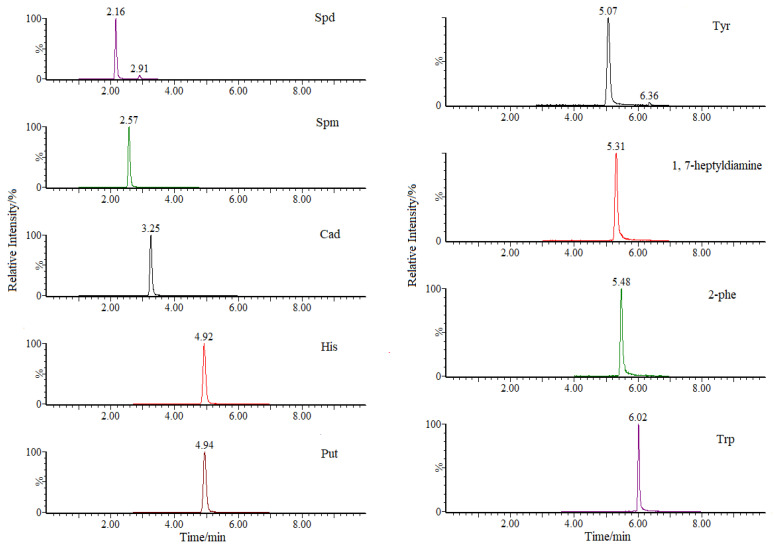
The multiple reaction monitoring (MRM) chromatograms for target compounds.

The eight BAs tested in this study are all small nitrogenous organic compounds with good water solubility and strong polarity, resulting in poor retention on the reverse-phase column. Two representative polar columns, including Waters ACQUITY UPLC BEH HILIC (100 × 2.1 mm, 1.7 μm) and Waters ACQUITY HSS T3 (100 mm × 2.1 mm, 1.8 μm), were compared. According to the operating requirements of the two columns, acetonitrile was selected as the organic phase, and 5 mmol/L ammonium acetate and 0.1% formic acid in water were preliminary used as the aqueous phases, respectively. The separation results showed that Spm and Spd had poor peak shape on the HILIC column, regardless of the water phase and gradient elution scheme. In contrast, all eight BAs showed better selectivity and higher sensitivity on the Waters ACQUITY HSS T3 column, with symmetrical peak shapes of the BAs and 1,7-heptyldiamine. Therefore, the ACQUITY HSS T3 column was selected for further optimization. Using acetonitrile as the organic phase, the separation effects of 5 mmol/L ammonium acetate, 5 mmol/L ammonium acetate containing 0.1% formic acid and 0.1% formic acid aqueous solution on BAs were compared. Subsequently, the elution gradient program, injection volume and flow rate were further optimized. Ultimately, acetonitrile and 5 mmol/L ammonium acetate containing 0.1% formic acid were chosen as the mobile phase. The MRM chromatograms of the target compounds are shown in Figure 2, and the retention times of eight BAs are presented in Table 1. The optimized gradient elution conditions are detailed in Section 2.2.

### 3.2. Characterization of Fe_3_O_4_@C-NFs

**Figure 3:**
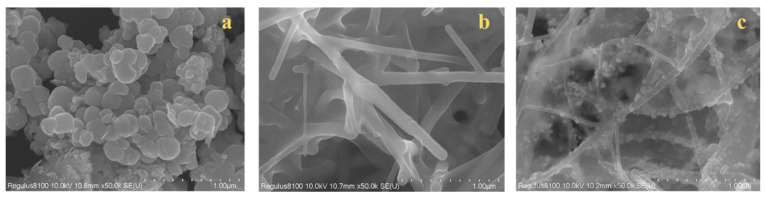
SEM images of Fe_3_O_4_ (**a**), C-NF (**b**) and Fe_3_O_4_@C-NF nanocomposites (**c**).

The surface morphology of Fe_3_O_4,_ C-NF, and Fe_3_O_4_@C-NF composites was investigated using SEM, as shown in Figure 3. The SEM images revealed that Fe_3_O_4_ particles were uniformly distributed on the fiber rods of C-nanofibers, and the Fe_3_O_4_@C-NFs composites exhibited a large surface area with numerous sorption sites. Brunauer–Emmett–Teller (BET) surface area and total pore volume of the composites were 30.7 cm^2^/g and 0.30 cm^3^/g, respectively, indicating that the Fe_3_O_4_@C-NF composites have large specific surface area and a uniform pore radius distribution. Further characterization results for these materials can be found in our previous studies [40].

### 3.3. Selection of Extraction Solvent

**Figure 4:**
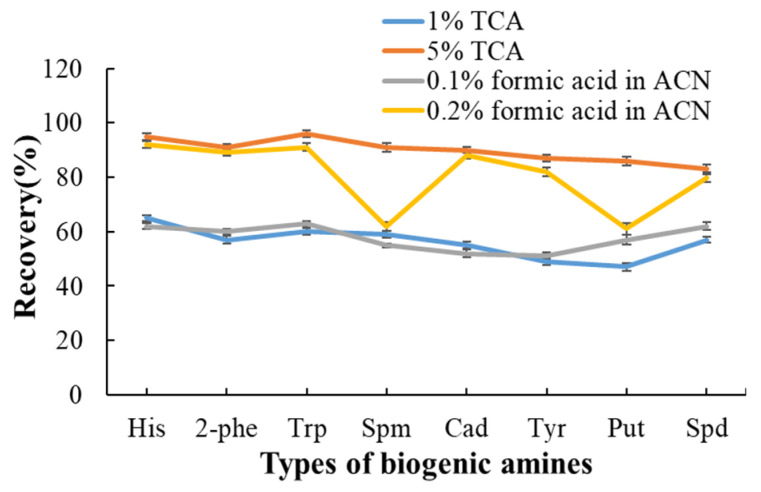
Effect of extraction solution type on recovery of 8 BAs.

The extraction solvents commonly used for biogenic amines (BAs) in aquatic products include trichloroacetic acid (TCA) [28], perchloric acid (HClO_4_) [29] and acetonitrile [42] in previous studies. Aquatic products contain impurities, such as protein, fat, mineral elements, etc.; therefore, a suitable extraction solution should not only efficiently extract BAs but also effectively precipitate proteins. BAs are mainly water-soluble small molecules. Spm and Spd are insoluble in acetonitrile. HClO_4_ is highly corrosive and harmful to the human body, which led to the exclusion of both acetonitrile and HClO_4_ from our study. Aqueous solutions of TCA at varying concentrations (1%, 5%) and acetonitrile with different proportions of formic acid (0.1%, 0.2%) were evaluated for extraction. Fresh *portunculus triverruca* purchased from local market served as the biological sample, to which a specific volume of a mixed standard BA solution was added. Recoveries were evaluated to examine the efficiency of different extraction solvents. Recoveries were determined by spiking the samples with the eight BA mixed standard solutions and calculated as measured content vs. fortification level. As illustrated in Figure 4, extraction efficiencies using a 1% TCA solution and 0.1% formic acid in acetonitrile (*v*/*v*) resulted in low yields for each BA. The extraction efficiencies using 0.2% formic acid acetonitrile (*v*/*v*) yielded poor results for Spm and Put. In contrast, better extraction efficiencies for all eight BAs were achieved using 5% TCA solution, which was ultimately selected as the final extraction solvent. Given that BAs have low molecular weights and high water solubility, acidic reagents can effectively interact with proteins to form insoluble salt precipitates, thereby reducing interference. The concentration of 1% TCA was insufficient for effective protein precipitation, while a 5% TCA solution achieved extraction efficiencies exceeding 80% for all eight BAs.

### 3.4. Optimization of Adsorption and Elution Conditions for Biogenic Amines by Magnetic Materials

Several parameters, including sample solution pH, adsorption and desorption time, adsorbent usage, type and volume of eluent, were investigated in this study.

The adsorbent Fe_3_O_4_@C-NFs is a highly interwoven and branched mesoporous material with a large specific surface area, which facilitates the adsorption of biogenic amines (BAs) through hydrogen bonding under specific conditions.

**Figure 5:**
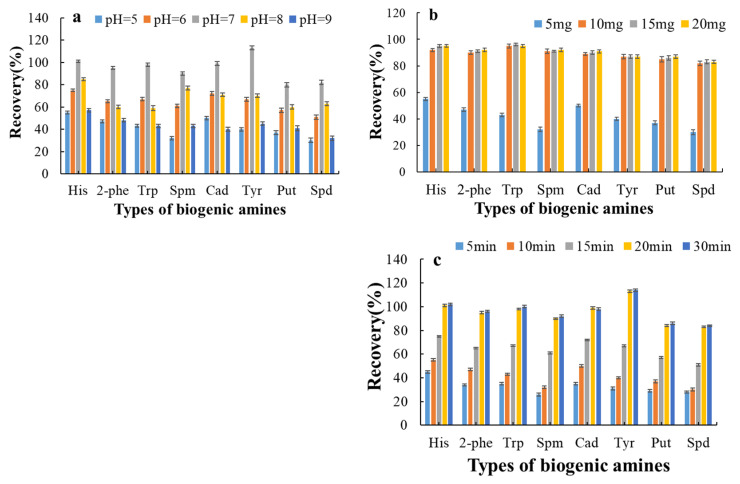
Optimization of SMPE conditions for 8 BAs. (**a**) Effect of pH, (**b**) adsorbent usage, (**c**) adsorption time.

The impact of pH on the recovery of eight BAs was evaluated, with the results illustrated in Figure 5a. The recovery of the eight BAs increased gradually as the pH increased from 5 to 7, reaching a maximum at pH 7, followed by a significant decline with further increases in pH. The pH level can affect the stability of adsorbents and their surface binding sites, as well as the chemical properties of the target analytes, thereby influencing the magnitude of the interactions between the two. BAs are bound to magnetic materials in their molecular form, and the amino group in BAs can form hydrogen bonds with hydroxyl groups on the adsorbent surface in a neutral environment, resulting in strong adsorption [43]. However, under acidic or alkaline conditions, the -NH_2_ group in BAs may become protonated, or the hydroxyl groups on the adsorbent surface may become ionized, which can hinder hydrogen bond formation and reduce adsorption efficiency. Based on these findings, a pH of 7.0 was selected as the optimized pH for subsequent experiments in this work.

To optimize the usage of the adsorbent, varying amounts ranging from 5 to 20 mg (5, 10, 15 and 20 mg) were tested. As shown in Figure 5b, the extraction recoveries of BAs increased as the amount of adsorbent rose from 5.0 mg to 10.0 mg, with no significant improvement observed beyond 20 mg of adsorbent. The results demonstrated that 10.0 mg of adsorbent could achieve optimized extraction performance for the eight BAs under given conditions.

Various adsorption times ranging from 5 to 30 min were investigated. As shown in Figure 5c, the extraction efficiencies gradually increased with time, reaching a plateau at 20 min, beyond which no significant changes were observed. This indicated that a rapid distribution equilibrium between BAs and the adsorbent was established within 20 min.

Different elution solvents were tested, including acetonitrile with varying proportions of formic acid (2%, 5%, 10%, *v*/*v*) and different proportions of acetonitrile–water (9:1, 7:3, 5:5, *v*/*v*) containing 5% formic acid. The results indicated that BAs could be effectively desorbed using 5% formic acid in acetonitrile–water (9:1, *v*/*v*).

### 3.5. Method Validation

The proposed method’s linearity, accuracy, precision, sensitivity and matrix effects were evaluated under optimized experimental conditions. Linearity was conducted at six concentration levels, corresponding to 2, 5, 10, 50, 100, 200 ng/mL for 2-Phe, Tyr, Trp and 20, 50, 100, 500, 1000, 2000 ng/mL for His, Spd, Spm, Cad, Put, all containing 100 ng/mL of the internal standard. Calibration curves were generated using the internal standard (IS) method, which involved plotting the quantitative ion peak area ratios of each BA to the internal standard against the corresponding BA concentrations. Favorable linearities for BAs were obtained, with correlation coefficients (r^2^) exceeding 0.995. Additionally, the individual residual deviations were below 20% for each calibration standard. Limits of detection (LODs) were established at 3-times the signal-to-noise ratio, while limits of quantitation (LOQs) were set at 10-times this ratio. For His, Spd, Spm, Cad and Put, the LOD and LOQ were 1.0 mg/kg and 3.0 mg/kg, respectively; for 2-Phe, Tyr, Trp, the LOD and LOQ were 0.1 mg/kg and 0.30 mg/kg, respectively. A sample of *Portunus trituberculatus* was chosen for accuracy and precision assessments. Intra-day precision was evaluated by analyzing spiked samples at three levels in five replicates within a single day, while inter-day precision was determined by analyzing samples over five consecutive days. The validation results, presented in Table 2, demonstrated favorable recoveries ranging from 84.7% to 115%. Intra-day and inter-day precisions fell within the range of 3.7% to 7.5% and 4.2% to 7.7%, respectively, indicating acceptable method repeatability and reproducibility. Matrix effects were measured by comparing the peak areas of analytes added post-extraction with pure BA standard solutions prepared in the mobile phase. Matrix effects were 89.0–99.7%, which are well within acceptable ranges (<20%). These findings suggest that the established method exhibits favorable accuracy and precision for analyzing the BA content in crabs.

### 3.6. Reproducibility and Recyclability of Fe_3_O_4_@C-NF Composites

For the sake of reproducibility, Fe_3_O_4_@C-NF composites prepared in different batches were utilized for MSPE, as outlined in Section 2.4. The inter-batch precisions were determined by subtracting the method precision from the relative standard deviations (RSDs) of the analyte content. The calculated inter-batch precisions fell within a range of 4.8–7.5%, indicating that Fe_3_O_4_@C-NF composites were prepared with good reproducibility. The reusability experiment was conducted by washing the adsorbent with adequate amounts of deionized water and methanol after each use and then reusing it for subsequent MSPE, as described in Section 2.4. There was no significant difference in extraction efficiency between newly synthesized and recycled composites. Based on the results obtained, the adsorbent can be reused at least seven times with less than 5% loss in extraction recoveries.

### 3.7. Comparison with Other Methods

The established method is compared with traditional pre-derivatization methods and some novel non-derivatization methods.

Derivatization reactions often present several challenges, including complex operational procedures, stringent derivatization conditions, instability of derivatized products, and poor method reproducibility. Additionally, the use of derivatization reagents poses health risks to laboratory personnel and contributes to environmental pollution. In this study, we opted not to use derivatization reagents to circumvent these issues. Compared to certain non-derivatization methods, our approach not only facilitates the successful application of carbon-based materials for the adsorption of biogenic amines but also allows for the recycling of these materials. This method exhibits more pronounced environmentally friendly characteristics than previously reported techniques, as it does not involve any harmful derivatization reagents. Furthermore, the adsorbent utilized in the MSPE was synthesized through a one-step method that does not require toxic or hazardous substances, making the synthesis process both green and straightforward. Moreover, the sensitivity and accuracy of our method are comparable to those of several non-derivatization approaches.

### 3.8. Changes in Biogenic Amines in Pickled Swimming Crabs Under Different Storage Conditions

**Figure 6:**
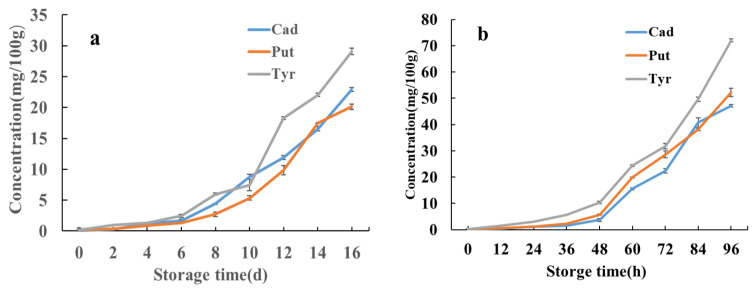
Changes in Cad, Put and Tyr of choking crab during storage at 4 °C and 20 °C (**a**), 4 °C; (**b**), 20 °C.

The effects of storage temperature (4 °C and 20 °C) and storage time on the contents of BAs in pickled swimming crabs were studied. The concentrations of eight BAs formed during storage are indicated in Table 3 and Table 4. The changes in Cad, Put and Tyr during storage are shown in Figure 6.

Among the BAs evaluated, His, Spd, Spm and 2-Phe were not detected during storage, while Cad, Put and Tyr were the predominant amines formed. These three BAs exhibited an increasing trend over the entire storage period, with a slow increase in the early stage followed by a significant increase in the later stage. During storage at 20 °C, Cad, Put and Tyr showed a slight increase from 0 to 36 h, followed by a sharp increase from 36 to 96 h, with Tyr demonstrating the highest rate of increase. The levels of Cad, Put and Tyr reached 47.1 ± 0.49, 52.3 ± 1.60 and 72.0 ± 0.57 mg/100 g, respectively, after 96 h. Similarly, during storage at 4 °C, Cad, Put, and Tyr exhibited a slight increase from 0 to 6 days, followed by a sharp increase from 6 to 16 days, with Tyr showing the most significant change, consistent with the trend observed at 20 °C. The levels of Cad, Put and Tyr reached 29.0 ± 0.56, 20.0 ± 0.45 and 29.0 ± 0.56 mg/100 g, respectively, after 16 d. These results demonstrated that low-temperature storage can obviously inhibit BA production. These findings illustrate that storing at low temperatures can effectively inhibit the production of BAs. The temperature exerts a significant influence on the generation of BAs within crab tissues. This phenomenon may be attributed to the heightened proliferation and reproduction of microorganisms with increasing storage temperature, resulting in a quicker transition to the logarithmic growth phase. This, in turn, leads to the abundant production of amino acid decarboxylase and protein-degrading enzymes, consequently yielding a substantial quantity of biogenic amines and free amino acids. The elevation in free amino acids accelerates the rate of biogenic amine production. Consequently, at elevated temperatures, there is a marked increase in both the production rate and content of biogenic amines.

The results of this study are consistent with previous reports. For instance, Laly et al. [44] identified Cad, Put, and Spd as the major amines produced during the spoilage of three-spotted crabs (*Portunus sanguinolentus*), with His being negligible throughout both storage methods. Cad, Put and Spd reached levels of 91.2 ± 2.6, 0.91 ± 0.07 and 31.1 ± 2.9 mg/kg on the 8th day of refrigerated storage and 106.1 ± 2.1, 72.1 ± 2.3 and 1.39 ± 0.05 mg/kg on the 12th day of iced storage; Similarly, His was not detected in the edible tissues of Chinese mitten crabs (*Eriocheir sinensis*) stored at 30 °C for 24 h, while Put and Cad were the predominant amines in another study [45]; Anupama et al. [46] also reported that His was detected at a very low level and did not vary significantly during storage, reaching 0.06 mg/kg on the rejection day (192 h). After 192 h of storage at 4 °C, Cad, Put, and Spd reached levels of 87.67, 0.88, and 37.6 mg/kg, respectively.

In contrast, the predominant BAs identified in this study differed from some previous investigations. Xu et al. [47] reported that His was the primary biogenic amine formed in Chinese mitten crab (*Eriocheir sinensis*) during storage, reaching peak levels of 91.22 mg/kg after 72 h at 4 °C and 181.23 mg/kg after 24 h at 20 °C, with Tyr, Trp, Put, and Cad all being present at levels below 10 mg/kg.

Arulkumar et al. [48] reported that His was the major biogenic amine formed, followed by Trp, Put, Tyr in blue swimmer crab (*Portunus pelagicus*) muscle during storage at both 4 °C and 20 °C after 96 h. Specifically, Put was detected at the highest concentrations of 7.25 ± 0.92 mg/100 g and 10.82 ± 0.23 mg/100 g after 96 h at 4 °C and 20 °C, respectively. Tyr was present at levels of 4.98 ± 0.49 mg/100 g at 4 °C and 125.6 ± 1.49 mg/100 g at 20 °C after the same duration. The storage of blue swimmer crabs at 4 °C significantly reduced the formation of tyramine compared to storage at room temperature, which aligns with our findings.

The findings of this study differed from previous research on crabs regarding the type or content of predominant BAs. This discrepancy may be attributed to variations in sample sources and treatment methods for the crabs, as well as differences in the types and relative abundances of BA-producing bacteria [49]. Some studies [44,45,46,50] have indicated that His is not consistently detected in all “decomposed” samples, while Put and Cad, which are responsible for the putrid odor of decomposed seafood, are recognized as significant spoilage markers in aquatic products due to their potential to exacerbate histamine toxicity [50,51].

Tyramine concentrations exceeding the maximum permissible levels of 10–80 mg/100 g can induce hypertension in both humans and animals [52]. Additionally, at 84 h, the total concentration of BAs at 20 °C reached 128.52 mg/100 g, significantly higher than the 75.59 mg/100 g found in samples stored at 4 °C. This concentration also exceeded the FDA guidelines for BA levels (100 mg/100 g) [53,54]. Lower storage temperatures contributed to the consistent quality of the pickled crab.

## 4. Conclusions

In the present work, a sensitive and effective method for determining eight types of BAs in crabs was established using UPLC–MS/MS without derivatization. By utilizing magnetic covalent adsorption material, the extraction process of the method was simplified and the purification step was accelerated. The method was demonstrated to have good linearity, high accuracy and precision, and obvious advantages over traditional derivative methods in terms of operation steps, analysis time and sensitivity. The method was successfully applied to determine the concentrations of eight BAs in salting *Portunus trituberculatus*, demonstrating its suitability for BA analysis in crabs.

This experiment systematically studied the variations in the levels of eight BAs in the tissue of salting *Portunus trituberculatus* meat stored at 4 °C for 16 days and 20 °C for 96 h. Throughout the storage duration at both refrigeration and room temperature, the levels of Cad, Put, and Tyr exhibited an increasing trend, with these three biogenic amines showing significant differences during storage (*p* < 0.05). Cad, Put, and Tyr were found to be produced at a faster rate during storage at 4 °C compared to storage at 20 °C.

## Figures and Tables

**Table 1:** Conditions of multiple reaction monitoring for 8 biogenic amines and the internal standard.

Analyte	RT (min)	Parention Ion (*m*/*z*)	Daughter Ion (*m*/*z*)	Cone Voltage (V)	Collision Energy (eV)
His	4.92	111.9	95.1 *, 68	15	15, 22
2-phe	5.48	121.9	105 *, 77	15	10, 25
Trp	6.02	160.9	122 *, 77	10	10, 22
Spm	2.57	202.9	112.1 *, 129	20	20, 15
Cad	3.25	102.9	86 *, 69	19	15, 22
Tyr	5.07	137.9	144.1 *, 117	13	10, 21
Put	4.94	88.9	72 *, 30	12	12, 20
Spd	2.16	145.9	112 *, 72	15	15, 15
1,7-heptyldiamine	5.31	130.9	114.1 *, 55.1	15	10, 15

* quantitative ion.

**Table 2:** Results of recovery and precision of spiked samples (*n* = 6).

Analyte	Spiked Levels (μg/kg)	Accuracy (%)	Intra-Day RSD (%)	Inter-Day RSD (%)	Analyte	Spiked Levels (μg/kg)	Accuracy (%)	Intra-Day RSD (%)	Inter-Day RSD (%)
His	2.0	84.7	6.0	5.6	Cad	2.0	112	7.0	5.9
	10.0	85.5	6.3	6.2		10.0	95.2	6.0	4.2
	50.0	91.6	7.2	5.8		50.0	93.5	4.9	2.2
2-phe	2.0	88.3	5.7	6.7	Tyr	2.0	90.7	5.3	3.6
	10.0	91.5	4.9	5.9		10.0	89.6	6.8	4.7
	50.0	85.2	6.1	7.7		50.0	91.5	6.9	3.9
Trp	2.0	88.7	3.7	5.9	Put	2.0	92.8	7.1	4.0
	10.0	89.2	5.4	4.3		10.0	93.0	5.2	5.9
	50.0	90.1	4.7	4.7		50.0	115	5.9	5.2
Spm	2.0	88.9	6.9	5.3	Spd	2.0	92.4	6.6	4.3
	10.0	91.1	7.0	6.2		10.0	97.3	7.5	5.7
	50.0	90.7	5.7	5.3		50.0	88.1	6.2	4.1

**Table 3:** Changes in biogenic amine content in *Portulus trisulatus* during storage at 4 °C.

BAs mg/100 g	Storage Time/d							
	2	4	6	8	10	12	14	16
Spd	ND	ND	ND	ND	ND	ND	ND	ND
Spm	ND	ND	ND	ND	ND	ND	ND	ND
Cad	0.284 ± 0.018	1.24 ± 0.0275	1.70 ± 0.1785	4.40 ± 0.121	8.80 ± 0.43	11.9 ± 0.2875	16.5 ± 0.3485	22.9 ± 0.346
His	ND	ND	ND	ND	ND	ND	ND	ND
Put	0.306 ± 0.0045	0.876 ± 0.0125	1.35 ± 0.01	2.37 ± 0.37	5.36 ± 0.35	9.85 ± 0.7825	17.5 ± 0.065	20.1 ± 0.450
Tyr	0.976 ± 0.00115	1.36 ± 0.0115	2.47 ± 0.254	5.98 ± 0.1495	7.42 ± 0.94	18.3 ± 0.25	22.1 ± 0.25	29.0 ± 0.560
2-Phe	ND	ND	ND	ND	ND	ND	ND	ND
Trp	ND	ND	ND	ND	ND	ND	ND	ND

ND: not detected.

**Table 4:** Changes in biogenic amine content in *Portulus trisulatus* during storage at 20 °C.

Bas mg/100 g	Storage Time/h								
	0	12	24	32	48	60	72	84	96
Spd	ND	ND	ND	ND	ND	ND	ND	ND	ND
Spm	ND	ND	ND	ND	ND	ND	ND	ND	ND
Cad	0.117 ± 0.0105	0.42 ± 0.0015	1.09 ± 0.0205	1.48 ± 0.10	3.71 ± 0.515	15.6 ± 0.3575	22.5 ± 0.8555	40.8 ± 1.871	47.1 ± 0.4855
His	ND	ND	ND	ND	ND	ND	ND	ND	ND
Put	0.180 ± 0.0015	0.572 ± 0.0095	1.23 ± 0.01	2.30 ± 0.04	5.37 ± 0.17	19.9 ± 0.16	28.7 ± 1.28	38.1 ± 0.49	52.3 ± 1.595
Tyr	0.214 ± 0.0205	1.50 ± 0.0215	3.13 ± 0.0925	5.71 ± 0.0625	10.3 ± 0.4475	24.3 ± 0.408	31.7 ± 1.009	49.65 ± 0.8595	72.0 ± 0.566
β-Phe	ND	ND	ND	ND	ND	ND	ND	ND	ND
Trp	ND	ND	ND	ND	ND	ND	ND	ND	ND

ND: not detected.

## Data Availability

The data that support the findings of this study are available from the corresponding author upon reasonable request.

## Abbreviations

  Tyr, Tyramine; 2-Phe, 2-phenylethylamine; His, Histamine; Trp, Tryptaminen; Spd, Spermidine; Spm, Spermine; Cad, Cadaverine; Put, putrescine; MSPE, Magnetic solid-phase extraction; Fe_3_O_4_@C-NFs, C-nanofiber-coated magnetic nanoparticles; BAs, Biogenic amines.
